# Multi-scale organization in communicating active matter

**DOI:** 10.1038/s41467-022-34484-2

**Published:** 2022-11-07

**Authors:** Alexander Ziepke, Ivan Maryshev, Igor S. Aranson, Erwin Frey

**Affiliations:** 1 Arnold Sommerfeld Center for Theoretical Physics and Center for NanoSciences, Ludwig-Maximilians-Universität München, Theresienstraße 37, 80333 Munich, Germany; 2 Department of Biomedical Engineering, Pennsylvania State University, University Park, PA 16802 USA; 3 Max Planck School Matter to Life, Hofgartenstraße 8, 80539 Munich, Germany

**Keywords:** Nonlinear phenomena, Cellular motility, Biological physics

## Abstract

The emergence of collective motion among interacting, self-propelled agents is a central paradigm in non-equilibrium physics. Examples of such active matter range from swimming bacteria and cytoskeletal motility assays to synthetic self-propelled colloids and swarming microrobots. Remarkably, the aggregation capabilities of many of these systems rely on a theme as fundamental as it is ubiquitous in nature: communication. Despite its eminent importance, the role of communication in the collective organization of active systems is not yet fully understood. Here we report on the multi-scale self-organization of interacting self-propelled agents that locally process information transmitted by chemical signals. We show that this communication capacity dramatically expands their ability to form complex structures, allowing them to self-organize through a series of collective dynamical states at multiple hierarchical levels. Our findings provide insights into the role of self-sustained signal processing for self-organization in biological systems and open routes to applications using chemically driven colloids or microrobots.

## Abstract (web summary)

The communication in active systems plays an important role in their self-organization, yet the detail is not fully understood. Here, Ziepke et al. show the formation of complex structures at multiple scales amongst interactive agents that locally process information transmitted by chemical signals.

## Introduction

Active matter encompasses a broad class of non-equilibrium systems that transduce energy stored in the environment into mechanical motion. In its most common form, locally interacting, self-propelled agents form coherent collective states that exceed the size of a single agent by orders of magnitude. Examples range from a variety of biological systems such as swimming bacteria^1–3^, cytoskeletal motility assays^4–6^, swarms, and flocks and schools of larger animals^7^, to synthetic self-propelled colloids^8,9^ and swarming microrobots^10,11^. There is broad agreement that self-propulsion, local alignment, and random disorientation of simple agents are fundamental microscopic determinants that can explain the occurrence of large-scale collective behavior.

However, in addition to local short-range interactions, such as alignment and collisions, many biological and synthetic systems exhibit various types of long-range signaling strategies. The social amoeba *Dictyostelium discoideum* uses cell-to-cell cyclic adenosine monophosphate (cAMP) concentration waves and chemotaxis to induce aggregation under harsh conditions^12,13^, insects rely on sound to coordinate the formation of cohesive swarms^14^, protein waves control cargo transport^15^, some active colloids form oscillating clusters using long-range chemical Ag/AgCl coupling^16,17^, microrobots and robotic fish use infrared, electrical and acoustic signals to communicate^18,19^. Signal transduction allows organisms to develop successful survival techniques that give them an evolutionary advantage over non-communicating organisms^20,21^. Communication facilitates the emergence of novel dynamic steady states, such as large streams and localized vortices^13^. Without communication, such states are not generic and are observed only under specific boundary conditions, particle chirality, or density-dependent feedback mechanisms^22,23^. Despite its importance, the role of communication in the context of active matter remains largely unexplored.

A significant body of literature focuses on self-propelled particles with diffusive (chemotactic) interactions. Studies on chemotactic colloids report on the formation of localized clusters and colliding polar bands, both established through motility-induced phase-separation (MIPS)^24–26^. There, the chemical interactions between different agents are mostly linear and passive, e.g., with a constant emission of the signal by the individual agents^27,28^. Distinct from these earlier studies, we ask about the role of an active, non-trivial agent’s response (decision-making) to detected signals. The information processing and decision-making should enable the complex hierarchical organization akin to living matter that does not occur in systems with passive chemical signaling.

To reveal the fundamental role of interparticle communication for self-organization, we chose to study a system of self-propelled units (agents) with local polar-alignment interactions. In addition, each agent can perform a specific task, namely, to detect and relay a signal transmitted between agents. Inspired by social amoebae that use cyclic adenosine monophosphate (cAMP) for communication^29^, and Gram-negative bacteria that employ acyl-homoserine-lactone (AHL) molecules as quorum-sensing signals^30,31^, we consider agents that broadcast a signal in the form of a chemical substance into the environment, where it spreads diffusively. Once the local level of the signal exceeds a certain threshold, agents tend to produce and propagate it. Thus, the agents act like a Schmitt trigger, a simple nonlinear electronic circuit with hysteresis^32^. Such a signal transduction system constitutes a spatially extended excitable medium that generically exhibits spiral waves of signaling activity. These waves can control the spatial self-organization of the agents by entraining their direction of self-propulsion. Thus, unlike existing models of amoeboid or bacterial aggregation^33–37^, self-propelled motion, rather than Brownian motion, is the primary mode of transport in our system. In contrast to Vicsek-type models^38^, the model incorporates the ubiquitous signaling found in biological systems. It thus provides insight into specific behaviors such as aggregation in social amoebae^39^ and oscillatory colloids^16^ and sheds light on the fundamental properties of active matter consisting of agents with “on-board” signal processing capabilities. The combination of chemical communication and internal information processing leads to an aggregation process involving collective dynamic states at multiple scales. We identify the decision-making machinery of the individual active agents as the driving mechanism for the collectively controlled self-organization of the system.

## Results

### Model

**Fig. 1:**
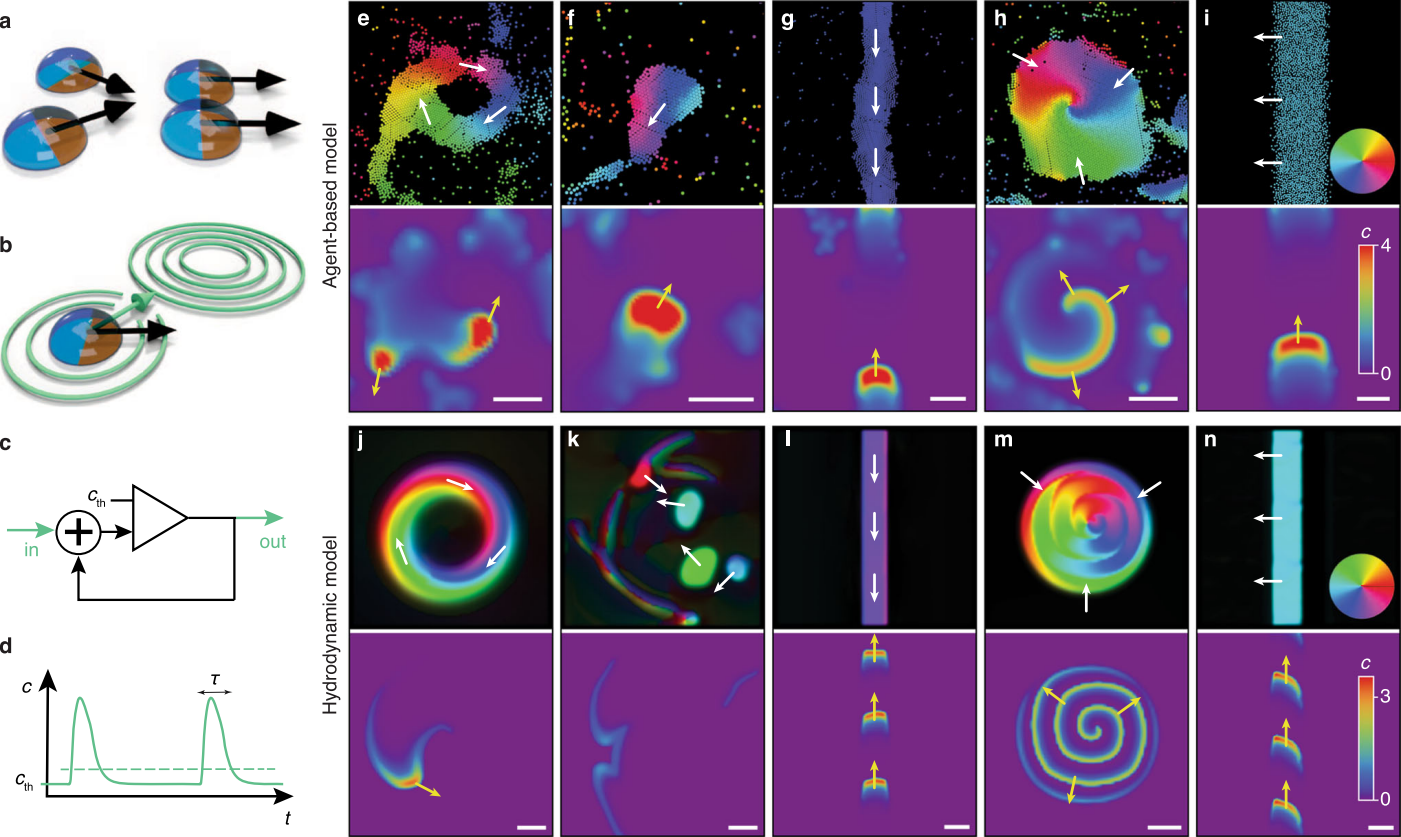
Schematics of the agent-based model for communicating active matter and summary of collective dynamic states. **a** Polar self-propelled particles undergo alignment in binary collisions. **b** A diffusible signal (green) aligns the cells’ orientation vectors. **c** Schematic of a Schmitt trigger with variable threshold *c*_th_. **d** Temporal response *c(t)* of the agents’ signaling system with characteristic timescale $\tau$. **e**–**n** Representative collective dynamic states in the agent-based (**e**–**i**) and the hydrodynamic model (**j**–**n**). The snapshots illustrate aggregation and vortex formation following initial ring formation (**e**, **j**), where remnant spiral wave arms induce chemical wave propagation in the ring after the spiral core vanished due to depletion in its center (‘whispering gallery’-modes); active droplets (**f**, **k**), with a collective response to external stimuli; a collective stream (**g**, **l**), where agents propagate toward the source of communication waves; a large vortex with a spiral wave (**h**, **m**), and a polar band (**i**, **n**). White scale bars indicate a length of 10 units. Colors indicate the polar orientation of particles (top panels) and the chemical concentration *c* (bottom panels). White and yellow arrows illustrate the direction of motion of the particles (top panels) and the propagation direction of signaling activity (bottom panels), respectively. Parameters are defined in Supplementary Note 3.

We consider an agent-based description of communicating active matter, in which each agent moves with velocity ${{{{{\bf{v}}}}}}={v}_{0}\,{{{{{\bf{n}}}}}}$ and is endowed with signal detection and relaying capability whose activity depends on an internal state variable *s*. The dynamics of the agents’ positions **r**_*i*_ *=* (*x*_*i*_*,y*_*i*_)^T^ is described by

1 $$\frac{d{{{{{{\bf{r}}}}}}}_{i}}{{dt}}={v}_{0}\,{{{{{{\bf{n}}}}}}}_{i}+\mathop{\sum }_{j\left[{r}_{{ij}} < 2{r}_{p}\right]}{f}_{{ij}}$$

where ${{{{{{\bf{n}}}}}}}_{i}={\left({{\cos }}{\varphi }_{i},{{\sin }}{\varphi }_{i}\right)}^{{{{{{\rm{T}}}}}}}$ is the unit vector in the direction of the *i*-th agent’s orientation *φ*_*i*_, with *i* = 1,…, *N*; *N* is the total number of agents in the domain. While the speed ${v}_{0}$ of each particle is assumed to be constant, their direction of motion **n** can change—owing to inelastic binary collisions that favor polar alignment (Fig. 1a) or in response to a chemical signal (Fig. 1b). Within an interaction radius ${r}_{{{{{{\rm{c}}}}}}}$, agents align in a polar fashion, i.e., the interaction of an agent with a neighbor causes both agents to turn toward the average orientation angle with the alignment rate $\varGamma$. If agents approach each other below a critical distance $2{r}_{{{{{{\rm{p}}}}}}}$, they obey a hard-core repulsion interaction cast as an isotropic short-range force ${f}_{{ij}}$ between the agents in Eq. (1). Akin to chemotaxis, the agents align with a certain sensitivity $\omega$ along the concentration gradient ${\varphi }_{c}={{{\tan }}}^{-1}\big({\partial }_{y}c/{\partial }_{x}c\big)$ of the local maximum of the chemical signal concentration *c*. These competing alignment processes are generally error-prone, which is accounted for by a white-noise term ${\xi }_{i}$ with amplitude $\sqrt{2{D}_{{{{{{\rm{R}}}}}}}}$. Specifically, we assume that the dynamics of the agent’s orientation ${\varphi }_{i}$ over time *t* is given by the Langevin equation

2 $$\frac{{{{{{\rm{d}}}}}}{\varphi }_{i}}{{{{{{\rm{d}}}}}}t}=-{\varGamma} \mathop{\sum }_{j[{r}_{ij} < {r}_{{{{{{\rm{c}}}}}}}]}\frac{\sin ({\varphi }_{i}-{\varphi }_{j})}{|{{{{{{\bf{r}}}}}}}_{i}-{{{{{{\bf{r}}}}}}}_{j}|}+\omega \,\sin ({\varphi }_{c}-{\varphi }_{i})+{\xi }_{i},$$

incorporating binary inelastic collisions between neighboring agents with spatial distance ${r}_{{ij}}=\left|{{{{{{\bf{r}}}}}}}_{i}-{{{{{{\bf{r}}}}}}}_{j}\right|$, chemotactic reorientation of agents along the concentration gradient of chemical signaling molecules^40^, and noise, respectively. The orientation along chemical gradients is implemented similarly to the agents’ polar alignment with their neighbors. For instance, in social amoeba the ability of chemotaxis is stable over large ranges of concentrations and alignment can be assumed to be independent of the absolute signal strength^41^.

Signal detection and self-sustained relaying are modeled by a Schmitt trigger (Fig. 1c): if the signal amplitude (i.e., chemical concentration) is above some threshold value ($c > {c}_{{{{{{\rm{th}}}}}}}$), an agent in a quiescent state (${s}_{0}=0$) switches into an excited state (${s}_{{{{{{\rm{ex}}}}}}} > 0$), and over a period $\tau$ it broadcasts the signal (Fig. 1d), i.e., releases a certain amount of the chemical into the environment, where it diffuses (with diffusion constant ${D}_{c}$) and is also degraded with rate $\alpha$. This yields the chemical signal dynamics

3 $${\partial }_{t}c({{{\bf{r}}},\, t})={D}_{c}\Delta c-\alpha c+\beta \mathop{\sum }_{i=1}^{N}f\left(\left|{{{{{\bf{r}}}}}}-{{{{{{\bf{r}}}}}}}_{i}\right|\right)\phi \left({s}_{i},c\right),$$

with a Gaussian spatial source distribution *f*(|**r**|), Laplace operator $\Delta$, and temporal derivative ${\partial }_{t}$. The agents act as sources of the chemical signal as

4 $$\beta \phi \left({s}_{i},c\right)=\beta \left(1-{s}_{i}\right)\varTheta \left(c-{c}_{{th}}\right),$$

with Heaviside-type signal detection and production rate *β*. The threshold value *c*_th_ as well as the source strength depend on the internal state, whose dynamics, for simplicity, is assumed to be linearly adapting to the signal concentrations,

5 $$\frac{{{{{{\rm{d}}}}}}{s}_{i}}{{{{{{\rm{d}}}}}}t}=\epsilon \left(c-{s}_{i}\right).$$

The response of the agents’ state *s*_*i*_ to recent stimuli mimics adaptation of receptor sensitivity and productiveness of the signal-emission. Taken together, the model incorporates the fundamental ingredients of a system of self-propelled active matter capable of communication; see “Methods” for a more extensive description of the agent-based model. Exemplary aggregation dynamics of a system without active decision-making are studied in the Supplementary Note 2.

As a complementary approach based on this microscopic model, we derive a hydrodynamic theory formulated in terms of the agents’ density field $\rho \left({{{{{\bf{r}}}}}},t\right)$, the polarization field ${{{{{\bf{p}}}}}}\left({{{{{\bf{r}}}}}},t\right)$, the internal state variable $s\left({{{{{\bf{r}}}}}},t\right)$, and the concentration of the chemical signal $c\left({{{{{\bf{r}}}}}},t\right)$, all of which depend on the spatial position ${{{{{\bf{r}}}}}}$ and time $t$,

6 $${\partial }_{t}\rho \left({{{{{\bf{r}}}}}},t\right)=-{v}_{0}\nabla \cdot {{{{{\bf{p}}}}}}+{D}_{\rho }\Delta \rho,$$

7 $${\partial }_{t}{{{{{\bf{p}}}}}}\left({{{{{\bf{r}}}}}},\, t\right)=\sigma \left(\rho -1\right){{{{{\bf{p}}}}}}-\delta {\left|{{{{{\bf{p}}}}}}\right|}^{2}{{{{{\bf{p}}}}}}+{D}_{p}\Delta {{{{{\bf{p}}}}}}-\chi {{{{{\bf{p}}}}}}\cdot \nabla {{{{{\bf{p}}}}}}-Q\left(\rho \right)\nabla \rho+\rho \omega \nabla c,$$

8 $${\partial }_{t}c\left({{{{{\bf{r}}}}}},\, t\right)={D}_{c}\Delta c-\alpha c+\rho \beta \varTheta (c-{c}_{{{\rm{th}}}})\left(1-s\right),$$

9 $${\partial }_{t}s\left({{{{{\bf{r}}}}}},\, t\right)={D}_{\rho }\Delta s+\epsilon \left(c-s\right)-\bar{v}\,{{{{{\bf{p}}}}}}\cdot \nabla s.$$

The hydrodynamic model comprises a coupled set of partial differential equations for these fields with basically the same parameters as the agent-based model (see “Methods” for details and Supplementary Note 1 for a derivation of the hydrodynamic theory from the agent-based model). In the absence of communication, e.g., $c\equiv 0$, the parameters *σ* and *δ* regulate the emergence of polar order above a mean-field critical density $\rho$_c_  = 1 when polar alignment interactions outweigh angular diffusion. Based on the large-scale field equations, we can study the dynamics of communicating active matter on length- and time-scales, not accessible with agent-based numerical simulations due to their high computational costs.

### Collective dynamic states

Communicating active matter exhibits unprecedentedly rich spatiotemporal dynamics and collective states, both during aggregation and in the final non-equilibrium steady state. The agent-based model and the hydrodynamic theory show that the emergence of order occurs through the hierarchical formation of distinct collective dynamic states (Supplementary Movie 1). These states include directed particle streams in which the agents move toward the source of chemical waves, ring-like streams with agents migrating along closed paths, compact motile droplets (active droplets), and large vortices that serve as sources of chemical spiral waves (Fig. 1e–n). The juxtaposition of the spatial organization of the particles (Fig. 1e–n, top panels) and the concentration field of the chemical signal (Fig. 1e–n, bottom panels) reveals a tight interdependence between the collective states of active matter and the chemical patterns.

Each of the collective dynamic states has a specific dynamics and a degree of stability. Vortices are well-localized and are stabilized by spiral waves trapped inside these dense aggregates. Their polarization vector **p** is oriented perpendicular to the outer vortex boundary and points inward, preventing agents from escaping and, therefore, stabilizing the vortex (Fig. 1h, m). While vortices are stable and robust, ring-like particle streams (Fig. 1e, j), retained by “whispering-gallery” waves, are long-lived but metastable and are typically engulfed by neighboring vortices (Supplementary Movie 9). Active droplets (Fig. 1f, k) lack an intrinsic source of excitable waves, and their direction of migration is generally determined by external signal gradients. They dissolve in the absence of guiding stimuli. A particle stream (Fig. 1g, l) can be considered a limiting case of a ring-like stream (with an infinite radius of curvature and planar signaling waves). It establishes an efficient collective long-distance particle transfer toward the source of the signaling waves. Finally, we also observe bands resembling the polar bands that develop in non-communicating Vicsek-like models^5^ (Fig. 1i, n). However, if agents in polar bands are coupled to chemical signaling waves propagating along the bands, as shown in Fig. 1i, n, this will induce a change of the agents’ orientation and may lead to a transition toward stream-type solutions as depicted in Fig. 1g, l.

Given these phenomenological observations, we ask two fundamental questions: How can different collective dynamic steady states be selected by tuning characteristic properties of the particle dynamics and the communication process? How can one characterize the hierarchical self-organization process and quantify the information processing involved?

**Fig. 2:**
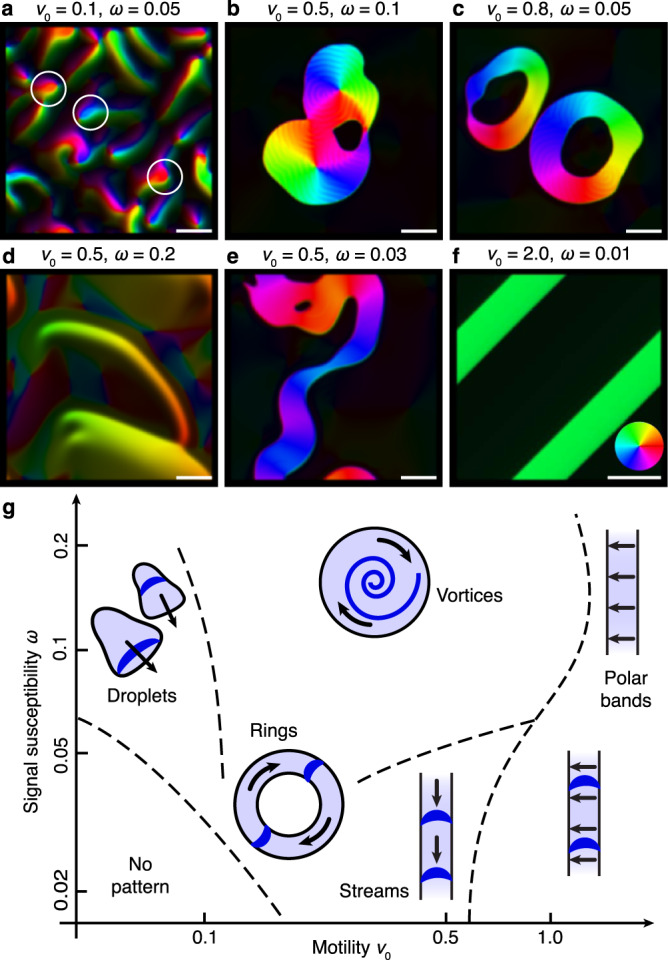
Principal collective dynamic states in the hydrodynamic model. The phase diagram of dominant (meta-stable) dynamic states in the $\omega -{v}_{0}$ (signal susceptibility and motility) parameter space is shown in the lower panel **g**, and snapshots of corresponding numerical simulations of the hydrodynamic model, starting from a homogeneous initial density ${\rho }_{0}$ = 0.6 and random initial excitations of the signaling system are depicted in the upper panels. Colors indicate the polar orientation within the aggregates. **a** Active droplets (three are highlighted by white circles), **b** vortex states, **c** ring solutions, **d** “silent” polar bands, **e** streams, **f** polar bands with signaling activity. See Supplementary Movies 2–7. The polar relaxation rate is set to *σ* = 0.02, remaining parameters are given in Supplementary Note 3. White bars indicate a length of 50 units.

Figure 2 shows the (qualitative) phase diagram with the representative collective dynamic states as a function of the agents’ mobility and signal sensitivity. In contrast to the isotropic-polar transition in Vicsek-type systems at $\rho=1$^42^, order here emerges at much lower densities, depending on the signal sensitivity (Supplementary Figure 1). This is due to the alignment of the polar particles with the collectively established signaling field. At a given particle density, the dominant collective dynamic state in the asymptotic non-equilibrium steady state is determined by the relative fraction of motility and signaling effects. Vortices and rings are the dominant structures in a parameter regime with low motility and high signaling sensitivity. Thereby, vortices exhibit a balance between the persistent self-propulsion promoting agents away from the localized vortices and chemotactic attraction toward the vortex’ center due to persistent spiral wave activity in the signaling field. If self-propulsion outweighs the attractive force established by collective signaling, vortices split up, and ring-like states become the predominant solution. If self-propulsion is rather weak and dominated by diffusion effects, the steady-state is governed by active droplets. Conversely, for vanishing signal sensitivity, the model reduces to a Vicsek-type model^38^, and only polar bands are found. These can either host persistent signaling activity or remain in the quiescent state of the signaling machinery, just like system-spanning polar bands in Vicsek-like models.

Next, we asked how the hierarchical aggregation process from a disordered arrangement of particles to the final non-equilibrium steady state can be understood based on our characterization of the various collective dynamic states (Fig. 1). To this end, we focus on a parameter regime with intermediate polarity relaxation times and a balance between motility and signaling effects, which ultimately gives rise to vortex states.

### Hierarchical self-organization

**Fig. 3:**
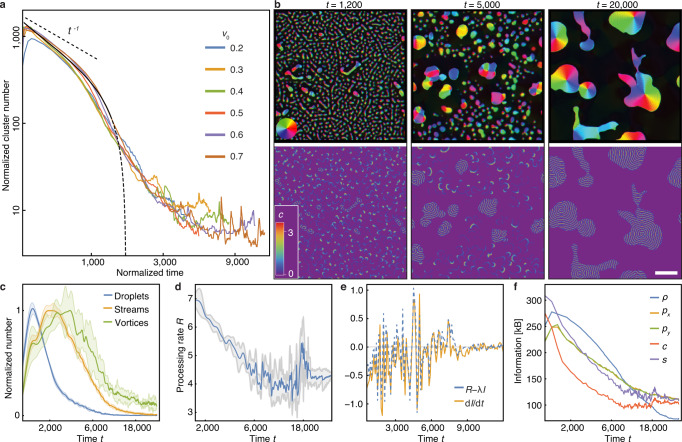
Hierarchical self-organization and information processing. **a** Time dependence of the cluster number *N*_c_ for different values of the mobility parameter ${v}_{0}$ in rescaled quantities $t\cdot {v}_{0}$ and ${N}_{{{{{{\rm{c}}}}}}}/{\sqrt{v}}_{0}$. The unlabeled black line indicates the estimate ${N}_{{{{{{\rm{c}}}}}}} \sim ({N}_{0}-\kappa {t}^{2})/t$. **b** Simulation snapshots at time *t*, displaying droplet ripening, vortex-controlled aggregation, and merging of vortices. The scale bar indicates a length of 100 units. Colors indicate polar orientation (top panels) and signaling concentration (bottom panels), respectively. **c** Time-resolved classification of collective dynamic states averaged over six simulation runs; the lighter shades define intervals of standard deviations. Initially, droplets grow and aggregate to form streams and vortices. **d** Time evolution of the information processing rate *R* of the signaling system and standard deviations (gray) averaged over six simulation runs. **e** Comparison of the rate of change ${{{{{\rm{d}}}}}}I/{{{{{\rm{d}}}}}}t$ of the stored information as predicted from Eq. (10) (blue) and the temporal derivative of compressed file sizes (orange). **f** Time dependence of the information content of the various fields, Eqs. (6)–(9). Parameters are *ω* = 0.05 and the values given in Supplementary Note 3. Panels **b**–**f** show simulation results and analysis for ${v}_{0}=0.5$. See “Methods” for details.

Our agent-based simulations and numerical integration of the hydrodynamic theory consistently show that the hierarchical self-organization process is facilitated by an intricate interplay of self-propulsion, signaling, and information processing (Fig. 3, Supplementary Movie 8). Initially, small-scale density fluctuations form, out of which droplets, streams, and small clusters later emerge. These initial aggregation processes are facilitated by short distance signaling waves and a local mutual entrainment. At later stages, the aggregation is orchestrated by spiral waves of signaling activity. Interestingly, there is competition between the spiral waves: Those that occupy larger and denser areas (mounds) accordingly have a higher frequency and displace smaller spiral waves (Supplementary Figure 2). As a result, higher particle density provides a positive feedback mechanism that favors the formation of larger aggregation centers^43^. The aggregation stage is characterized by competition between particle clusters, which is quite different from that of non-signaling active matter [e.g., motility-induced phase separation (MIPS)], where the number of clusters scales as ${N}_{{{{{{\rm{c}}}}}}}\sim {t}^{-\eta }$ with $\eta \approx 2/3$^44^. In our hydrodynamic model, we observe multi-scaling behavior, indicating qualitatively distinct types of processes (Fig. 3a, b) for the time evolution of the cluster number and the density and polarization fields. Initially, we observe ${N}_{{{{{{\rm{c}}}}}}}\sim 1/t$ (Fig. 3a), consistent with interface-controlled Ostwald ripening of clusters^45^. Once the streams have formed, there is a qualitative change in the aggregation process. The aggregation rate is now limited by the persistent directed motion of clusters and streams which migrate toward the aggregation centers. This leads to a much faster decay of the cluster number, even compared to the ballistic coalescence of clusters which would correspond to $\eta=2$. This ‘streaming phase’ is followed by the formation of a few localized vortices that contain most agents. Due to the low particle density in between the vortices and the resulting lack of signal transmission, the interaction between these structures is strongly attenuated, and the coarsening process is slow. Since the signaling field decays exponentially (with diffusion length ${L}_{{{{{{\rm{c}}}}}}}\sim \sqrt{{D}_{c}/{{{{{\rm{\alpha }}}}}}}$), one expects a logarithmic coarsening law ${N}_{{{{{{\rm{c}}}}}}}\sim 1/{{{{{\rm{ln}}}}}}t$^46^, consistent with the slow decay seen in our numerical data (Fig. 3c).

Thus, the ability to process information and make decisions results in the radically different organization of polar active matter. Ordering begins below the threshold of the polar-isotropic transition. The process leading to the formation of large vortices as robust attractors in the final stage of aggregation is much faster than that observed in non-signaling active matter or active matter with passive chemical signaling^24^. This is because it can exploit multi-scale collective intermediate states, whose respective frequencies are quantified in Fig. 3c. This classification confirms the observed phenomenology. The initial phase is dominated by coarsening of droplets. Once organizing vortices emerge, they establish persistent signaling waves. This causes a rapid decrease in the number of droplets and induces progressive aggregation through the formation of streams toward the vortices. In the final phase, a slow coarsening process occurs among the vortices with a corresponding logarithmic decrease in their number.

### Information processing drives self-organization

Since each agent is endowed with a decision-making capability, we also sought to characterize the course of information processing during the multi-scale hierarchical aggregation process. To this end, we quantified the time evolution of the information content $I\left(t\right)$ in the system, using the computable information content of a lossless compressed configuration of the physical fields $c$, $\rho$, ${{{{{\bf{p}}}}}}$, and $s$^47,48^. In particular, we consider the file sizes obtained by the Lempel-Ziv-Welch compression algorithm^49^ as implemented in the PNG file format (see “Methods”). The system’s information content changes over time as individual agents process information in response to external stimuli employing their self-propulsion and intrinsic signal processing capability (Schmitt triggers). In the absence of signaling, self-propulsion and local interactions are unable to create order at subcritical densities due to dominant angular diffusion; accordingly, the information content will decline exponentially with some decay rate $\lambda$ as the system approaches the disordered homogeneous state. Here, however, there is information processing which leads to self-organization and induces order. We quantify the information processing by the rate $R$ of agents transitioning to the refractory state, i.e., agents that emit a signal in response to a stimulus and therefore process information (Fig. 3d). Altogether, we expect the system’s information content to follow the dynamics

10 $$\frac{{dI}}{{dt}}\sim R-\lambda I,$$

with a fitting parameter $\lambda$. That, in turn, implies that the temporal change in the stored information depends exclusively on the initial information content and the measured processing rate $R.$

The basic hypothesis, Eq. (10), is validated by our numerical simulations (Fig. 3e). On a qualitative level, it agrees very well with the predicted evolution of information content. In particular, the prediction captures not only the overall trend but also coincides with important landmark points of the evolution. This affirms our assertion that the signaling machinery is key for information processing and the driving mechanism behind self-organization in the system.

The rate of change of the encoded information approaches a final state in which the order generated by persistent signaling offsets the loss of correlation created by the agents’ self-propulsion.

An analysis of the amount of information stored in the various fields also reveals the different stages of the aggregation process (Fig. 3f). We observe that the amount of information stored in the density field decreases and eventually approaches a comparably low value once the mass has accumulated in only a few stable vortices. This reflects the results of the cluster number analysis (Fig. 3a), including the qualitative change in aggregation dynamics between the dominance of ripening and the dominance of the guided movement of active droplets. In contrast to the homogeneous density field, the information content of the polarity field saturates at higher values, which correlates with the presence of persistent vortex states. Interestingly, the information content of the chemical concentration field $c$ exhibits a super-exponential decay. This confirms that information processing is mainly performed by the signaling machinery, which makes it an essential factor in the organization of the aggregation process. Moreover, it approaches its baseline information level earlier than the density field, indicating the transition toward the phase of nearly isolated vortex states.

## Discussion

In conclusion, we have introduced a new class of active matter equipped with self-sustaining signaling capabilities: it allows self-propelled agents to communicate and process information. Communication and decision-making enable hierarchical self-organized aggregation to emerge via a sequence of distinct collective dynamic states.

While our model is generic and does not rely on specific biological or chemical details, the observed phenomenology closely resembles the aggregation dynamics of social amoebae, including the formation of stable vortices^13^ and metastable rings^50^. Communication induces a non-trivial self-organized attraction that gives rise to the formation of a rich variety of collective dynamic states. The exhibited behavior in terms of collective dynamic states and the collectively controlled aggregation process is a clear advance compared to current models of chemotactic colloids. Besides the variety of observed states, communication and active information processing introduce a new framework of collective organization. It allows for much faster aggregation times and a controlled competition between aggregation centers as high-density clusters can enlarge their basin of attraction.

There are several potential extensions to the model, such as locally coupled self-propelled relaxation oscillators, signaling nematic active matter, or self-propelled agents coupled via sound or electromagnetic waves, which may have direct relevance to technological applications such as self-organizing swarms of minimal drones or functional microrobots. Information processing could be introduced by modifying the chemistry of colloids and droplets, thus allowing experimentally accessible realizations to be directly established for silver-chloride Janus colloids exhibiting chemical oscillations and synchronization^16,17^, and for self-propelled emulsions hosting the Belousov-Zhabotinsky reaction^51^, to name but two. Decision-making can also be implemented using simple electronic circuits in mass-manufactured microrobots. These may open new avenues for applications of active matter in nanoscience and robotics.

## Methods

### A detailed description of the agent-based model

In the agent-based model, we consider self-propelled particles with radius *r*_p_ in a two-dimensional square periodic domain with side length *L*. The particles move with constant speed *v*_0_ in the plane. The dynamics of the agents’ positions **r**_*i*_ is described by Eq. (1). The direction of movement can be changed by polar alignment during collision events, chemotactic responses to signaling molecules, or stochastic fluctuations. If two agents come within a distance of less than $2{r}_{{{{{{\rm{p}}}}}}}$, they are repositioned according to the following hard-core repulsion rule: overlapping particles are shifted in the direction of their distance vector by equal amounts until a distance of $2{r}_{{{{{{\rm{p}}}}}}}$ is restored. Within an interaction radius ${r}_{{{{{{\rm{c}}}}}}} > 2{r}_{{{{{{\rm{p}}}}}}}$, agents align in a polar fashion, i.e., the interaction of an agent *i* with a neighbor *j* causes both agents to turn toward the average orientation angle with the alignment rate $\varGamma$. The agents also align with the direction ${\varphi }_{c}={{{\tan }}}^{-1}\left({\partial }_{y}c/{\partial }_{x}c\right)$ of the local maximum of the chemical signal concentration *c* with the susceptibility coefficient $\omega$. Both alignment interactions are imperfect, which we account for by adding zero-mean white noise ${\xi }_{i}$ with amplitude $\sqrt{2{D}_{{{{{{\rm{R}}}}}}}}:\left\langle {\xi }_{i}\left(t\right){\xi }_{j}\left({t}^{{\prime} }\right)\right\rangle=2{D}_{{{{{{\rm{R}}}}}}}{\delta }_{{ij}}\delta \left(t-{t}^{{\prime} }\right)$. In total, the dynamics of the agent’s orientation ${\varphi }_{i}$ is given by the Langevin equation, Eq. (2).

The system of agents establishes self-sustaining chemical signaling as a means of information processing and transmission. Each agent is equipped with an internal state variable ${s}_{i}\in \left[{{{{\mathrm{0,1}}}}}\right]$ that determines whether or not it perceives the environment and transmits signals by emitting a chemical substance. We take the magnitude of *s*_*i*_ to be the refractoriness of an agent to external signals, i.e., a measure of how responsive it is to relay a signal: ${s}_{i}=0$ then corresponds to the state with the lowest refractoriness (highest susceptibility). The agents are assumed to sense the environment by linearly adapting to the local concentration level *c* of the chemical field with rate $\epsilon$, Eq. (3), and act as nonlinear sources of the chemical signal *c*. This release of chemicals depends on both the internal state of the agents and the environment. We assume the source strength to be of the threshold form, Eq. (4), where $\beta$ denotes the release rate and ${c}_{{{{{{\rm{th}}}}}}}$ a threshold above which agents can detect and relay signals and below which they remain quiescent; $\Theta \left(x\right)$ denotes the Heaviside step function with

11 $$\varTheta (x)\equiv \left\{\begin{array}{cl}1 &,\,{{{{{\rm{for}}}}}}\,x \, > \, 0,\\ 0 & \!\!\!\!\!\!\!\!\!\!\! ,\,{{{{{\rm{else}}}}}}.\end{array}\right.$$

The agent’s signaling receptors are assumed to undergo state-dependent changes in susceptibility that implement potential saturation effects and adaptation to varying levels of signaling molecules in the environment. Specifically, we take the threshold value ${c}_{{{{{{\rm{th}}}}}}}$ to be a linear function of the state variable ${s}_{i}$,

12 $${c}_{{{{{{\rm{th}}}}}}}\left({s}_{i}\right)=\frac{{s}_{i}+b}{a},$$

implementing a higher threshold for signal detection at larger state values of the refractoriness ${s}_{i}$. The parameter *b* sets the baseline threshold and the factor 1/*a* specifies the linear increase of the threshold ${c}_{{{{{{\rm{th}}}}}}}\left({s}_{i}\right)$ with growing state values. In addition, to implement the agents’ ability to process detected signals and respond to them, the release of chemicals shall depend on the internal state ${s}_{i}$ of an agent: In terms of their signal production, agents in the most susceptible state (${s}_{i}=0$) react most vigorously to super-threshold stimuli. The rate of signal release is assumed to decrease linearly ($1-s$) with increasing ${s}_{i}$. Note that for the set of parameters used in this study, Supplementary Note 3, the states *s*_*i*_ do not exceed values of one. Therefore, agents are always either quiescent and do not contribute to the chemical signaling field or act as sources for it.

Taken together, the interplay between the internal dynamics *s* and the chemical field *c* in a well-mixed environment is given by

13 $$\frac{{{{{{\rm{d}}}}}}s}{{dt}}=\epsilon \left(c-s\right),$$

14 $$\frac{{{{{{\rm{d}}}}}}c}{{{{{{\rm{d}}}}}}t}=-\alpha c+\beta \phi \left(s,c\right),$$

which also accounts for degradation of the emitted signal at a rate $\alpha$. Equations (13) and (14) constitute a nonlinear two-component system, which shows excitable behavior; see Supplementary Fig. 3a for an illustration of the phase-space flow. The quiescent state, corresponding to $c=s=0$, is linearly stable and has a finite domain of attraction. However, if for $s=0$ the input signal ${c}_{{{{{{\rm{in}}}}}}}$ exceeds the threshold ${c}_{{{{{{\rm{in}}}}}}} > {c}_{{{{{{\rm{th}}}}}}}\left(s=0\right)=b/a$, the system performs a long excursion in phase space before returning to $c=s=0$; see the red phase space trajectory in Supplementary Fig. 3a. Note that the amplitude of the response (extent of the red trajectory in phase space) is mainly determined by the phase-space flow and only weakly depends on the initial input strength ${c}_{{{{{{\rm{in}}}}}}}$. This ensures a sufficiently strong transmission of any super-threshold signals. The phase-space trajectory in Supplementary Fig. 3a yields the excitation pulse displayed in Supplementary Fig. 3b, which shows fast excitation and emission of signals and a slower refractory dynamics that restores the susceptible state ($c=s=0$). The duration of the refractory period $\tau$ is determined by the inverse of the relaxation rate ${\epsilon }^{-1}$.

Taken together, the excitable dynamics resemble the behavior of a Schmitt trigger (Fig. 1c), a circuit with closed negative feedback, which exhibits hysteresis-like dynamics representative of e.g., relaxation oscillators. In particular, the appropriate delay between the fast production of signaling molecules and the slower adaptation of the agent’s internal state can be achieved by choosing $\beta /\epsilon \gg 1$, resulting in a relaxation dynamics with a rapid response to a stimulus followed by a slower refractory period. Model parameters are summarized in Supplementary Note 3.

To formulate the spatial dynamics of the signaling molecules in terms of a concentration field *c*, one must specify how the molecules emitted by the agents are distributed in their vicinity. We use a source distribution given by a normalized Gaussian profile $f\sim {{\exp }}\left[-\left({x}^{2}+{y}^{2}\right)/\left(2{w}^{2}\right)\right]$ with characteristic width $w\equiv 2{r}_{{{{{{\rm{p}}}}}}}$. In addition, we account for the center-of-mass diffusion (with diffusion coefficient ${D}_{c}$) and degradation with rate $\alpha$, so that together with the source terms for each agent one obtains Eq. (3). We choose the decay rate $\alpha$ to be of the same order of magnitude as the positive source contributions, terms $\sim \beta$, to the signaling field *c* for average agent densities. On the scale of individual agents, signal diffusion is assumed to be fast compared to the agents’ self-propulsion velocity, $1\ll {D}_{c}/\left({r}_{{{{{{\rm{p}}}}}}}{v}_{0}\right)$. The parameters used in the numerical simulations are specified in Supplementary Table 1.

### A detailed description of the hydrodynamic model

In this section, we give a detailed description of the hydrodynamic model, Eqs. (6)–(9), that we introduced in the main text for communicating active-matter systems. This dynamic field theory is formulated in terms of a set of evolution equations for the following fields: the number density of particles $\rho$, the vector order parameter characterizing the particles’ local average polar alignment ${{{{{\bf{p}}}}}}=\left\langle {{{{{{\bf{n}}}}}}}_{i}\right\rangle$, the concentration of the signaling species *c*, and the state of refractoriness *s*. A representative vortex solution with internal spiral-wave activity of the signaling fields is shown in Supplementary Fig. 3a. We observe an approximately circular high-density cluster within which the particle orientation revolves around its center and aligns with the density gradients at the interface to the outer low-density regime. This vortex state is accompanied by the emergence of a spiral wave established inside the high-density domain by the chemical field and the adapting signaling states of the agents.

The time evolution of the agent’s density field $\rho \left({{{{{\bf{r}}}}}},t\right)$, Eq. (6), is given by an advection-diffusion equation, which accounts for advective transport due to the particles’ self-propulsion with speed ${v}_{0}$ and diffusion of the center of mass with diffusion constant ${D}_{\rho }$. The center-of-mass diffusion has no direct counterpart in the agent-based model as it has been neglected there. However, for completeness and to regularize density gradients, it is included in the hydrodynamic theory.

The direction of self-propulsion, described by the polar field ${{{{{\bf{p}}}}}}\left({{{{{\bf{r}}}}}},t\right)$, can be changed by interparticle interactions, stochastic fluctuations, and signaling-induced reorientations: The first three terms in Eq. (7) for the time evolution of the polarity field correspond to a time-dependent Ginzburg-Landau model describing the dynamics close to an isotropic-polar phase transition; units for the density $\rho$ are chosen such that the critical density is set to unity. The persistence parameter $\sigma$ defines the relaxation time, the parameter $\delta$ sets the magnitude of polar order, and ${D}_{p}$ implements the elasticity in a one-Frank-constant approximation. Moreover, to make the model more general, we include a term $\chi {{{{{\bf{p}}}}}}\cdot \nabla {{{{{\bf{p}}}}}}$ that accounts for self-advection. In the numerical simulations, the corresponding parameter $\chi$ is set to a small value and does not contribute critically to the qualitative behavior of the system. The coupling between the orientational order and density combines both self-advective and steric effects incorporated in the function

15 $$Q\left(\rho \right)=\frac{{v}_{0}}{2}\left[{\exp }\left(-32\rho \right)+{\exp }\left(16\left(\rho -2\right)\right)\right].$$

The steric effects can be modeled as an effective pressure. As derived in Supplementary Note 1, see Supplementary Eq. (11), we include the low-density contribution as an amplitude $Q\left(\rho \to 0\right)={v}_{0}/2$. For increasing densities, we assume that collective effects arising from particle interactions counteract the steric repulsion, and therefore reduce the amplitude of the function $Q\left(\rho \right)$. Complementing this, for high densities, the effective pressure contributions outweigh the collective effects again due to the finite volume of agents. Therefore, the amplitude $Q\left(\rho \right)$ increases at a critical maximum density of $\rho=2$. The coupling of the polar order to signaling encoded by the chemical concentration field enters in Eq. (7) via the term $\omega \nabla c$. It describes the alignment of the polarization field in the direction of the local maximum of the signal concentration *c* with susceptibility parameter $\omega$.

The dynamics of the chemical concentration field *c*, Eq. (8), is a direct transfer from the agent-based model, Eq. (3). Coarse-graining the equation, we replace the discrete sum of Gaussian source terms ∑_*i*_ *f* (|**r**−**r**_*i*_|) by a density-dependent continuous contribution $\sim \rho \left({{{{{\bf{r}}}}}},t\right)$.

The dynamics of the state variable *s*, Eq. (9), includes diffusive, reactive, and advective contributions. Here, the first term simply corresponds to the center-of-mass diffusion of the agents as in Eq. (6). The second term corresponds to the relaxation of the local state variable *s* to the corresponding local value of the signaling field *c*, where $\epsilon$ denotes the relaxation rate.

Therefore, the magnitude of the rate $\epsilon$ controls the timescale over which the internal signaling state *s* adapts to the chemical concentrations *c*. Finally, the term $\sim {{{{{\bf{p}}}}}}\cdot \nabla s$ incorporates the local change of the agents’ signaling states *s* by means of their self-propulsion. The regularizing prefactor $\bar{v}={{v_0\,}}{{\tanh }}\left(\left|{{{{{\bf{p}}}}}}\right|/\rho \right)/\left|{{{{{\bf{p}}}}}}\right|$ ensures the boundedness of effective self-propulsion velocities for low densities $\rho \to 0$.

### Numerical implementation

We integrate the agent-based model, Eqs. (1)–(5), on a square periodic domain with side length *L* over discretized time intervals $\Delta t$. For each time step, the continuous particle positions and orientations are updated following Eqs. (1), (2) and the hard-core repulsion rule, using an Euler-Maruyama scheme. For efficient identification of potential interaction partners at each time step, particles are assigned to virtual grid cells. We check for collisions within a particle’s grid cell and its surrounding cells. Agents that pass through a virtual grid cell’s boundaries are reassigned to their new grid cell. Based on the updated agent positions, we compute the agents’ source contributions, $\sim \beta$ to the continuous signaling field *c*. Subsequently, we solve the temporal dynamics of the signaling field, Eq. (3), in Fourier space by a forward Euler integration scheme and then obtain the representation in real space by inverse Fourier transform. We apply a fast Fourier transform algorithm for these transformations. Concluding the calculations for a given time, we update the internal states of the agents using the same forward Euler time integration scheme for Eq. (5). For the simulations with 4000 agents, shown in Fig. 1, we use a total system size of 200 × 200, resolved by 200 Fourier modes per spatial direction and a time step of $\Delta t=0.01.$ The depicted solutions are neither dependent on the selected spatial or temporal resolution which we verified by corresponding simulations with higher accuracy.

The set of continuous hydrodynamic Eqs. (6)–(9) is solved in a square periodic domain by a quasi-spectral method and a semi-implicit time integration with discretized time steps $\Delta t$.

For each time step, we make use of fast Fourier transform of the field quantities to calculate their spatial derivatives. Also, we compute the Fourier transform of the nonlinear contributions to the dynamics, and apply an exponential time differencing scheme of second order (ETD2) to integrate the complete set of equations in Fourier space over a time interval $\Delta t$^52^. In doing so, all linear contributions to the dynamics, Eqs. (6)–(9), are implicitly solved for, while nonlinearities are included explicitly via their first-order forward finite difference approximation. The eigenvalues and pseudoinverse of the matrix representation of the linear dynamics of Eqs. (6)–(9), necessary for ETD2, are calculated once at the beginning of the runtime using the linear algebra library Eigen^53^. We initialize the system with homogeneous densities and polarity fields with small zero-mean white noise perturbations. The chemical system is initialized by exciting randomly positioned and oriented two-dimensional Gaussian kernels of characteristic lengths ranging from 20 to 30 units and widths of 5 units. The model parameters are given in Supplementary Note 3. For all simulations, time steps and spatial resolutions have been adapted to optimize runtime while ensuring that results do not depend on the chosen discretization.

### Quantification of the aggregation process

The self-sustaining signaling mechanism we consider has a threefold effect on the formation and organization of large-scale structures in the active polar system. Firstly, signaling enables pattern formation from a homogeneous density, even below the critical density (${\rho }_{{{{{{\rm{c}}}}}}}\equiv 1$) for the polar ordering transition. Secondly, stronger chemotactic susceptibility of the polar orientation to the established signaling significantly increases the rate of the self-organization process, as can be seen in Supplementary Figure 1. Starting from an initially spatially uniform density ${\rho }_{0}$, the aggregation times ${T}_{{{\mbox{aggr}}}}$ for crossing the isotropic-to-polar ordering transition at ${\rho }_{{{{{{\rm{c}}}}}}}\equiv 1$ decrease significantly for larger signal susceptibilities $\omega$. And thirdly, spiral waves as sources of persistent signaling activity can stabilize the emerging vortex structures, as can be seen from the results of the numerical simulations, e.g., Fig. 3. To gain a better understanding of the principles underlying the signal-driven self-organization process and to quantify the degree and type of ordering, we use cluster classification analysis and quantify the time evolution of the information content in the system. Both methods are presented in more detail below.

In our numerical simulations, we observe that distinct collective states dominate the different phases of aggregation; see Fig. 3, Supplementary Movie 8. During an initial phase, droplets of agents are formed and undergo Ostwald-type ripening. Once spiral waves are established as persistent signaling sources, the droplets show directed motion toward the strongest of these sources, i.e., they become ‘active’ droplets. The coalescence of these active droplets leads to the formation of collective density streams. Eventually, streams and active droplets approach the source of the organizing signal, where they condense into stable clusters. The interplay of aggregation due to the intrinsic signaling and the self-propulsion of the polar active matter typically results in localized vortex solutions. As a means of classifying the various collective states discussed above, namely droplets, streams and vortices, we analyze clusters with densities $\rho > 0.7$ (above the system’s average density, which we typically set to ${\rho }_{0}=0.6$) by quantifying their total mass, spatial extension along their main axes, and the direction of the effective self-propulsion of the cluster. The latter represents the direction of the cluster’s center-of-mass motion, $\sim\int {{{\mathbf{p}}}} ({{{\mathbf{r}}}},\, t){{{\mathrm{d}}}}{{{\mathbf{r}}}}$. In particular, we measure the spatial extension of clusters along their main axes (axes with largest spatial extent), the angle between the main axis and the averaged cluster polarity, and the intrinsic vorticity $\nabla \times {{{{{\bf{p}}}}}}$ of the orientational field.

We classify a given aggregate as a stream if the shape factor (the ratio of major to minor diameter) is larger than 1.4 and the angle between the major axis and polarity is smaller than $\pi /4$; if the shape factor is less than 1.4 and the mean vorticity inside the domain exceeds a value of 0.01, the aggregate is classified as a vortex. Clusters characterized as neither streams nor vortices are classified as droplets. Information about domain position, orientation and shape is obtained by using the first three central moments of the binarized domain with density threshold $\rho=0.7$.

As a measure for emerging order in the system, and to quantify the impact of the signaling machinery on the aggregation process, we consider the total amount of information stored in the system. Following references^47,48^, the information content can be obtained by lossless compression of the system’s data, i.e., the data points of the discretized continuous fields, Eqs. (6)–(9), for a given time. We analyze the fields at discrete time points with step size $\Delta t=200$ for total simulation times of ${t}_{{{\mbox{sim}}}}={{{{\mathrm{40,000}}}}}$. In order to measure the information content of the system for a given time, we saved the data of all the separate fields into a collective image with a spatial discretization of 128 by 128 pixels per field and 256 gray values per pixel. Subsequently we use the lossless compression in the PNG format to compute the stored information content. The resulting file sizes then give a corresponding amount of stored information as discussed in the main text; see Fig. 3e, f. Information processing in the system is facilitated by two distinct processes: polar ordering due to pairwise collisions and decision-making of the individual signaling units, as specified by the excitable signaling field dynamics. Below the isotropic-to-polar transition at the critical density (${\rho }_{{{{{{\rm{c}}}}}}}=1$), the disordering effect of the agent’s angular diffusion dominates over their ordering alignment dynamics, such that in the absence of chemical signaling the system must relax toward a homogeneous disordered state. This relaxation process is expected to proceed at a rate $\lambda$. As an organizing factor, the signaling machinery counteracts the natural trend of the polar active-matter system toward the homogeneous state. We hypothesize that most of the information processing occurs through the signaling machinery, and we quantify its activity by the information processing rate *R*. The latter is represented by the area fraction of the excitable system in the refractory state. Specifically, we define this state as exhibiting a super-threshold concentration in the chemical signaling field, $c > 1$. Taken together, we posit that the time evolution of the stored information content *I* can be approximated as given in Eq. (10). By means of this dynamic equation, and based on the assumption that information in the system is mainly processed by the signaling machinery, we are able to predict the temporal evolution of the total stored information. Starting from a value of the system’s initial information content, and supplied with the time-dependent processing rates *R*, Eq. (10) allows for a prediction of the temporal dynamics of the stored information. The comparison between this prediction and the actual dynamics of the stored information content quantified by the file size of the lossless compressed data at a given time in Fig. 3e yields good agreement. This again validates the basic assumption of signaling-mediated information processing in the system.

Based on the cluster classification and cluster number analysis, we can quantify the three main stages of the aggregation process described above and in the main text; see also Fig. 3 and Supplementary Movies 1 and 8. In the following, we describe the basic modes of mass aggregation in terms of the efficiency of the processes. Consider a system of droplets of equal size, concentration *n* and diffusion coefficient $D\sim {S}^{{{{{{\rm{\gamma }}}}}}}$, with a yet-to-be determined exponent $\gamma$ relating the diffusion to the droplet sizes *S*. For diffusion-limited coalescence of droplets in two spatial dimensions, the time dependence of droplet sizes and numbers is given by^54^

16 $$S\sim {t}^{z},{N}_{c}\sim {t}^{-z},$$

where the exponent *z* can be determined from the hydrodynamic equations underlying the aggregation process at the corresponding stages. For instance, the probability of coalescence in a binary collision process is given by ${n}^{2}$, and thus, the mean-field equation for the droplet density *n* reads

17 $$\frac{{{{{{\rm{d}}}}}}n}{{{{{{\rm{d}}}}}}t}=-D\left(S\right){n}^{2}=-{D}_{0}{S}^{\gamma }{n}^{2}.$$

Substituting the expressions for *S* and *n*, one obtains for the exponent

18 $$z=\frac{1}{1-\gamma }.$$

For the case where diffusion does not depend on the cluster size, $\gamma=0$, one obtains ${N}_{{{{{{\rm{c}}}}}}}\sim 1/t$. This behavior is similar to the interface-controlled Ostwald ripening for which the coarsening of droplets is independent of their diffusive motion. In addition, our hydrodynamic model gives rise to directed motion of active droplets, which is guided by organizing spiral waves. Including the guided movement of active droplets toward the organizing vortices, one can estimate the cluster number dynamics by

19 $${N}_{c}\left(t\right)\sim \left({N}_{0}-\kappa {t}^{2}\right)/t,{{\mbox{ with}}}\,\kappa > 0.$$

This estimate incorporates the directed ballistic motion of clusters toward a collective aggregation center $\sim {N}_{0}-\kappa {t}^{2}$. Moreover, these clusters may still exhibit interface-driven coarsening, which is accounted for by an additional factor ${t}^{-1}$. Thus, the estimate captures the main behavior of the first two aggregation stages, which are dominated by Ostwald ripening and coordinated movement of droplets toward spiral waves as organizing centers. This becomes manifest in a good qualitative agreement between the estimate and the measured evolution of the cluster number as shown in Fig. 3a, with fit parameters ${N}_{0}={{{{\mathrm{382,000}}}}}$ and $\kappa=0.15$. However, at longer times, vortex-vortex competition, which is not accounted for in the given estimate, becomes increasingly important. Therefore, the deviations between the estimated and measured dynamics of the cluster numbers grow as the aggregation process progresses.

## Supplementary information

Supplementary Information

Description of Additional Supplementary Files

Supplementary Movie 1

Supplementary Movie 2

Supplementary Movie 3

Supplementary Movie 4

Supplementary Movie 5

Supplementary Movie 6

Supplementary Movie 7

Supplementary Movie 8

Supplementary Movie 9

## Data Availability

The data that support the findings of this study are available in the main text, methods, and supplementary information. Additional information is available from the corresponding authors upon request.

## Code availability

The code used in this study is available from the corresponding authors upon request.
