# Supplementary material for: Multi-scale organization in communicating active matter
Source: Nat Commun. 2022 Nov 7;13:6727. doi: 10.1038/s41467-022-34484-2 (PMC9640622; doi:10.1038/s41467-022-34484-2)
Supplement: Supplementary file 1 — Supplementary Information [file 41467_2022_34484_MOESM1_ESM.pdf]

Supplementary Information for

Multi-scale organization in communicating active matter

Alexander Ziepke,[1] Ivan Maryshev,[1] Igor S. Aranson,[2*] and Erwin Frey[1,3*]

[1]Arnold Sommerfeld Center for Theoretical Physics and Center for NanoSciences, Ludwig-Maximilians-Universität München, Theresienstr. 37, 80333 Munich, Germany
[2]Department of Biomedical Engineering, Pennsylvania State University, University Park, PA 16802, USA
[3]Max Planck School Matter to Life, Hofgartenstraße 8, 80539 Munich, Germany

*Correspondence to: isa12@psu.edu, frey@lmu.de

Supplementary Notes

1 Derivation of the hydrodynamic equations through a Boltzmann-like kinetic approach

In this section we show how the set of hydrodynamic equations,

$$\partial_t\rho(\boldsymbol{r},t) = -v_0\boldsymbol{\nabla}\cdot\boldsymbol{p} + D_\rho\,\Delta\rho\,, \tag{1a}$$

$$\partial_t\boldsymbol{p}(\boldsymbol{r},t) = \sigma\,(\rho-1)\,\boldsymbol{p} - \delta\,|\boldsymbol{p}|^2\,\boldsymbol{p} + D_p\,\Delta\boldsymbol{p} - \chi\,\boldsymbol{p}\cdot\boldsymbol{\nabla}\boldsymbol{p} - Q(\rho)\boldsymbol{\nabla}\rho + \rho\,\omega\boldsymbol{\nabla}c\,, \tag{1b}$$

$$\partial_t c(\boldsymbol{r},t) = D_c\,\Delta c - \alpha\,c + \rho\,\beta\,\Theta\,(c-c_{\mathrm{th}})\,(1-s)\;, \tag{1c}$$

$$\partial_t s(\boldsymbol{r},t) = D_\rho\,\Delta s - \epsilon\,(s-c) - \bar{v}\,\boldsymbol{p}\cdot\boldsymbol{\nabla}s\,, \tag{1d}$$

can be derived from a Boltzmann-like approach for the probability density $P(\boldsymbol{r},\varphi,t)$ of finding a particle at position $\boldsymbol{r}$ with orientation φ at time t; the particle's orientation is signified by the unit vector $\boldsymbol{n} = (\cos\varphi,\sin\varphi)^T$. The equation accounts for center-of-mass diffusion, particle self-propulsion, rotational diffusion, alignment with the signaling field, and interactions between particles:

$$\partial_t P(\boldsymbol{r},\varphi,t) = D_\rho\partial_i\partial_i P - v_0\,\partial_i(n_i P) + \partial_\varphi\big[D_{\mathrm{R}}\partial_\varphi + \omega(c)\sin(\varphi-\varphi_c)\big]P + \text{interactions}\,. \tag{2}$$

The advection term together with the rotational diffusion describe the self-propelled motion of the particles combined with the angular noise as in the agent-based model. The fourth term corresponds to a probability flux directed towards orientations that are aligned with the local gradients of the signaling field c with sensitivity parameter $\omega(c)$ and $\varphi_c \equiv \tan^{-1}(\partial_y c/\partial_x c) = \text{angle}\,(\nabla c)$. The interaction contributions will be discussed further below.

We follow the standard approach for deriving hydrodynamic equations from a Boltzmann-type of equation by expanding the probability density function in Fourier modes for the spatial orientation of the director $\boldsymbol{n}$ in two-dimensional space[1,2],

$$P(\boldsymbol{r},\varphi) = \sum_k P_k(\boldsymbol{r})\,e^{ik\varphi}\,, \tag{3}$$

whereby, for the sake of brevity, we suppress the time dependency here and in the following. The corresponding Fourier coefficients follow from the forward transform

$$P_k(\boldsymbol{r}) = \frac{1}{2\pi}\int_0^{2\pi}\mathrm{d}\varphi\,P(\boldsymbol{r},\varphi)\,e^{-ik\varphi}\,. \tag{4}$$

We define the particle density ρ and the density-weighted polar order $\boldsymbol{p}$ by relating them to the harmonics via the Fourier expansion, Eq. (3):

$$\rho(\boldsymbol{r}) \equiv \int_0^{2\pi}\mathrm{d}\varphi\,P(\boldsymbol{r},\varphi) = 2\pi P_0\,, \tag{5}$$

$$\begin{aligned}\boldsymbol{p}(\boldsymbol{r}) &\equiv \int_0^{2\pi}\mathrm{d}\varphi\,\boldsymbol{n}(\varphi)P(\boldsymbol{r},\varphi)\,,\\ &= \sum_k\frac{1}{2}\int_0^{2\pi}\mathrm{d}\varphi\,\Big(e^{i\varphi}+e^{-i\varphi}, i\left(e^{-i\varphi}+e^{i\varphi}\right)\Big)^T P_k(\boldsymbol{r})\,e^{ik\varphi}\,,\\ &= \pi\big(P_1+P_{-1}, i(P_1-P_{-1})\big)^T\,.\end{aligned} \tag{6}$$

To describe the intrinsic states of the communicating active matter, we introduce a probability density $P^s(s)$ of particles in a given signaling state s and assume that the total probability density $P_{\mathrm{tot}}(\boldsymbol{r},\varphi,s) = P^s(s)\,P(\boldsymbol{r},\varphi)$ factorizes in a part for the signaling state and the distribution for the agent's positions and orientations. Thus, the density-weighted signaling state of the agents is given by

$$\bar{s} \equiv \int \mathrm{d}s \int_0^{2\pi} \mathrm{d}\varphi\, s\, P^s(s)\, P(\boldsymbol{r},\varphi)\,. \tag{7}$$

In the following, the different contributions to the Boltzmann equation, Eq. (2), are analyzed separately. First, in order to derive expressions for the diffusive contributions in the hydrodynamic equations we use the projection onto the m-th harmonic,

$$\overline{(\ldots)}^{m} = \frac{1}{2\pi}\int_0^{2\pi} \mathrm{d}\varphi\, e^{-im\varphi}\,(\ldots)\,, \tag{8}$$

which gives the m-th Fourier coefficient to the expansion above, Eq. (3). Applying the projection operator, Eq. (8), onto the corresponding term in Eq. (2) one obtains

$$\partial_t \rho = D_\rho \Delta \rho\,, \tag{9}$$

for the dynamics of the density. One would obtain the same dynamics for the center-of-mass diffusion in the polar order field, but contributions from interaction kernels, representing elasticity of the polarity field, can lead to similar terms, which is why we assume a different coefficient D_p for the polar field. Continuing with the advective term, (i.e. $\sim v_0$), the projection onto the modes yields

$$\begin{aligned}
\partial_t P_m(\boldsymbol{r}) &= \overline{-v_0 \partial_i (n_i P(\boldsymbol{r},\varphi))}^{m}\,,\\
&= -\frac{v_0}{2\pi}\int_0^{2\pi} d\varphi \sum_k P_k(\boldsymbol{r}) e^{ik\varphi}\left[\partial_x e^{-im\varphi}\frac{(e^{i\varphi}+e^{-i\varphi})}{2} + \partial_y e^{-im\varphi}\frac{(e^{i\varphi}-e^{-i\varphi})}{2i}\right],\\
&= -\frac{v_0}{2}\left[\partial_x \sum_k P_k(\boldsymbol{r})(\delta_{k,m-1}+\delta_{k,m+1}) + i\partial_y \sum_k P_k(\boldsymbol{r})(\delta_{k,m+1}-\delta_{k,m-1})\right].
\end{aligned} \tag{10}$$

With the definitions, Eqs. (5) and (6), we obtain for the field variables

$$\partial_t \rho(\boldsymbol{r}) = 2\pi \partial_t P_0(\boldsymbol{r}) = -v_0 \partial_i p_i(\boldsymbol{r})\,, \tag{11}$$

$$\partial_t p_x(\boldsymbol{r}) = \pi \partial_t (P_1(\boldsymbol{r}) + P_{-1}(\boldsymbol{r})) = -\frac{v_0}{2}\partial_x \rho(\boldsymbol{r})\,, \tag{12a}$$

$$\partial_t p_y(\boldsymbol{r}) = i\pi \partial_t (P_1(\boldsymbol{r}) - P_{-1}(\boldsymbol{r})) = -\frac{v_0}{2}\partial_y \rho(\boldsymbol{r})\,. \tag{12b}$$

Since a Boltzmann-approach is by design a low-density approximation, these results must be interpreted as such and require for an extension to assure well-behavedness at higher densities. Notably, this applies to the coupling of the polarity field to density gradients, $\partial_t p_i \sim -\frac{1}{2}\, v_0\, \partial_i \rho$. At low densities, this term accounts for an effective pressure, increasing with increasing particle densities. At higher densities, other cooperative effects emerging

from anisotropic interactions can dominate the coupling of the polarity field to density gradients, counteracting the repulsion dominating at low densities. In addition, at a critical maximum density, which we set to $\rho = 2$, the effective pressure increases significantly due to the finite volumes of the agents. Therefore, steric interactions dominate the cooperative interactions for $\rho \to 2$. We account for these effects by extending the terms $\sim -\partial_i \rho$ by a density-dependent prefactor $Q(\rho)$ which is proportional to v_0 and has the following form:

$$Q(\rho) = \frac{v_0}{2}\Big[\exp\left(-32\rho\right) + \exp\left(16(\rho-2)\right)\Big] . \tag{13}$$

The function $Q(\rho)$ captures the repulsion at low densities which decays for intermediate densities due to cooperative effects. Moreover, it limits the maximum density to values $\rho \approx 2$ taking into account the steric repulsion at dense packing of the agents. The presented results do not qualitatively depend on the particular choice of the function $Q(\rho)$. The scalar field corresponding to the agent's signaling activity, Eq. (7) is directly associated with the agents. Hence, in the same way as the particles it is advected with the polar flow and exhibits center-of-mass diffusion. From the definition, Eq. (7), we obtain

$$\begin{aligned}
\partial_t \bar{s} &= -v_0 \int \mathrm{d}\varphi\, \mathrm{d}s\, s \partial_i n_i P \, , \\
&= -v_0 \int \mathrm{d}\varphi\, \mathrm{d}s\, s \left[\partial_x \frac{e^{i\varphi}+e^{-i\varphi}}{2} + \partial_y \frac{e^{i\varphi}-e^{-i\varphi}}{2i}\right] \sum_k P^k P^s \, , \\
&= -2\pi v_0 \int \mathrm{d}s\, s \left[\frac{1}{2}\, \partial_x \left(P^\varphi_{-1} + P^\varphi_1\right) + \frac{1}{2i}\, \partial_y \left(P^\varphi_{-1} - P^\varphi_1\right)\right] P^s \, ,
\end{aligned}$$

and with the definition of the polarity field, Eq. (6),

$$\partial_t \bar{s} = -v_0 \partial_i \left(\frac{\bar{s}\, p_i}{\rho}\right) . \tag{14}$$

Thus, the complete diffusive and advective contributions to the dynamics of the density weighted signaling state $\bar{s} = \rho s$ are given by

$$\partial_t \bar{s} = D_\rho \Delta \bar{s} - v_0 \partial_i \left(\frac{\bar{s} p_i}{\rho}\right) . \tag{15}$$

Correspondingly to the agent-based model, we re-express the state field $\bar{s}$ in terms of the 'state concentration', i.e., the local state normalized by the particle density, s by replacing $s = \bar{s}/\rho$ in Eq. (15); one obtains

$$\partial_t s = D_\rho \Delta s - \frac{v_0 p_i}{\rho} \cdot (\partial_i s) \, , \tag{16}$$

where we neglected cross-gradient contributions in the density ρ and the field s.

Next, we turn to the contribution of the angular noise to the dynamics of the polar field. Fourier-expanding the corresponding term $\sim D_\mathrm{R}$ in Eq. (2) and projecting it onto the jth harmonic according to Eq. (8), yields the equation

$$\partial_t P_j(\boldsymbol{r}) = -D_R j^2 P_j(\boldsymbol{r}) \tag{17}$$

and, thus, with the definition of the polar field, Eq. (6),

$$\partial_t \boldsymbol{p}(\boldsymbol{r}) = -2D_\mathrm{R}\, \boldsymbol{p}(\boldsymbol{r})\,. \tag{18}$$

Finally, regarding the alignment of the agents' orientation vectors with gradients of the signaling field c, we want to briefly highlight the origin of the corresponding terms, $\sim \omega$, in the Boltzmann equation (2) starting from the proposed underlying Langevin dynamics

$$\begin{aligned} \frac{\partial \boldsymbol{r}}{\partial t} &= v_0\, \boldsymbol{n}(\varphi)\,, \\ \frac{\partial \varphi}{\partial t} &= \xi(t) + \omega(c)\sin\left(\varphi - \varphi_c\right)\,, \end{aligned} \tag{19}$$

with the particle position vector $\boldsymbol{r}$ and the angle of the chemical gradient $\varphi_c = \mathrm{angle}(\nabla c)$. The chemotaxis contributes to the Boltzmann equation, Eq. (2), directly as the angular drift term

$$\partial_t P \sim -\partial_\varphi \left[\omega(c)\sin\left(\varphi - \varphi_c\right)\right] P\,. \tag{20}$$

Expanding the probability density in the Fourier harmonics as in Eq. (3), one obtains

$$\partial_t P_k = -\frac{\omega(c)}{2\pi}\int_0^{2\pi} \mathrm{d}\varphi\, e^{-ik\varphi}\partial_\varphi \left[\sin\left(\varphi - \varphi_c\right)\sum_{k'} P_{k'} e^{ik'\varphi}\right]\,, \tag{21}$$

and after integration by parts

$$\begin{aligned} \partial_t P_k &= -\frac{ik\omega(c)}{2\pi}\sum_{k'}\int_0^{2\pi} \mathrm{d}\varphi\, \left[\cos(\varphi_c)\sin(\varphi) - \sin(\varphi_c)\cos(\varphi)\right] P_{k'} e^{i(k'-k)\varphi}\,, \\ &= -\frac{ik\omega(c)}{4\pi}\sum_{k'}\left[\cos(\varphi_c)\left(i\delta_{k,k'-1} - i\delta_{k,k'+1}\right) - \sin(\varphi_c)\left(\delta_{k,k'-1} + \delta_{k,k'+1}\right)\right] P_{k'}\,. \end{aligned} \tag{22}$$

Using the definitions, Eqs. (5),(6), and neglecting contributions of the second harmonics, the response of the dynamics of $\boldsymbol{p}$ to the signaling stimulus is given by

$$\partial_t p_i = \omega\, \rho\, \partial_i c\,, \tag{23}$$

where we chose a linear dependence of the alignment strength on the signaling amplitude c, namely $\omega(c) = 4\pi\, \omega c$. The contributions arising from particles' interactions can be motivated as done in Refs.[3–5]. As such, we include for completeness an elasticity like contribution

$$\partial_t \boldsymbol{p} \sim D_p \Delta \boldsymbol{p}\,, \tag{24}$$

and a self-propulsion

$$\partial_t \boldsymbol{p} \sim \chi\, \boldsymbol{p}\cdot\nabla \boldsymbol{p}\,, \tag{25}$$

in the model. Both terms may arise from anisotropic interactions, e.g., for elongated particles. They are not included in the agent-based model and we set the corresponding parameters

D_p and χ to small values as the effects are not crucial for the reported behavior of signaling active matter. Altogether we obtain the set of hydrodynamic equations

$$\partial_t \rho(\boldsymbol{r}, t) = -v_0 \boldsymbol{\nabla} \cdot \boldsymbol{p} + D_\rho \, \Delta\rho \,, \tag{26a}$$

$$\partial_t \boldsymbol{p}(\boldsymbol{r}, t) = \sigma\,(\rho - 1)\,\boldsymbol{p} - \delta\,|\boldsymbol{p}|^2\,\boldsymbol{p} + D_p\,\Delta\boldsymbol{p} - \chi\,\boldsymbol{p}\cdot\boldsymbol{\nabla}\boldsymbol{p} - Q(\rho)\boldsymbol{\nabla}\rho + \rho\,\omega\boldsymbol{\nabla} c\,, \tag{26b}$$

$$\partial_t c(\boldsymbol{r}, t) = D_c\,\Delta c - \alpha\,c + \rho\,\beta\,\Theta\,(c - c_{\mathrm{th}})\,(1 - s)\;, \tag{26c}$$

$$\partial_t s(\boldsymbol{r}, t) = D_\rho\,\Delta s - \epsilon\,(s - c) - \bar{v}\,\boldsymbol{p}\cdot\boldsymbol{\nabla} s\,, \tag{26d}$$

complementing the derived contributions from the Boltzmann equation, Eq. (2), with the interaction terms, Eqs. (24), (25), and the continuous versions of the equations for the signaling machinery, Eqs. (3), (5) in the main text.

2 Reduced model without decision making

To highlight the role of the individual decision making for the multi-scale aggregation process, for comparison we also investigate the behavior of a system lacking such a mechanism. In particular, we modify the source dynamics given in main text Eq. (3), such that it becomes independent of the agents' internal state,

$$\partial_t c(\boldsymbol{r}, t) = D_c \Delta c - \alpha c + \beta \sum_{i=1}^{N} f(\boldsymbol{r}, t)\,. \tag{27}$$

The polar agents with dynamics given by main text Eqs. (1), (2), and supplementary information Eq. (27), are assumed to contribute as persistent sources of the signaling field. Similar to what has been reported in reference[6], we observe aster-like stationary cluster formation with interface controlled ripening, see Supplementary Fig. 4a. Moreover, the interplay between self-propulsion and attraction towards a local aggregation center can give rise to short-lived ring-like structures and vortices which eventually tend to dissolve into a few aster-like aggregates as depicted in Supplementary Fig. 4b. Since in the modified model there is only local interactions mediated by the comparably slow diffusion of the signaling field, it does not exhibit a collective long-range organization of aggregation centers. In contrast to a system with active decision making, here the established smaller aggregates collide and merge upon random encounters.

3 Model parameters

The supplementary tables 1-3 provide an overview of the system parameters used in the numerical simulations shown in the main text as well as in the supplementary figures and movies. We measure densities in units of the critical density for the isotropic-polar transition. Time is given in units of the signal decay rate $[\alpha]$ and lengths are given in units of the resulting diffusion length $\sqrt{D_c/[\alpha]}$.

Supplementary Tables

Parameter	Description	Value (continuum model)	Value (agent-based)
α	signal decay rate	10	0.9
β	signal production rate	40	2
a	threshold factor	0.9	0.9
b	constant threshold	0.05	0.05
ϵ	refractory rate	4	0.3
D_c	signal diffusion	1	0.9

Supplementary Table 1: Parameters of the signaling system, Eqs. (1c), (1d), in the excitable regime used for the hydrodynamic- and the agent-based model, respectively.

Parameter	Description	Value
v_0	propulsion speed	0.2
D_{R}	rotational diffusion	0.05
r_c	interaction radius	2
r_p	particle radius	0.25
Γ	polar alignment factor	0.1

Supplementary Table 2: Parameters of the agent-based model as detailed in Methods. The chemical susceptibility parameter in main text Fig. 1 is set to $\omega \in \{0.1, 0.4, 0.004, 0.2, 0.004\}$ for panels e-i, respectively.

		Value				
Parameter	Description	Default	Fig. 1j	Fig. 1k	Fig. 1 l,n	Fig. 1 m
v_0	motility	0.5	0.1	0.5	0.2	0.1
σ	polar persistence parameter	0.01	0.1	0.2	0.5	0.05
ω	signal susceptibility	0.1	0.8	0.3	0.8	0.4
D_ρ	translational diffusion	0.05				
D_p	elasticity parameter	0.1				
χ	convective derivative coefficient	0.1				
δ	magnitude of bulk order	1.0				
ρ_0	average density	0.6				

Supplementary Table 3: Parameters of the hydrodynamic continuum model, described in Methods. Figure numbers correspond to main text figures.

Supplementary Figures

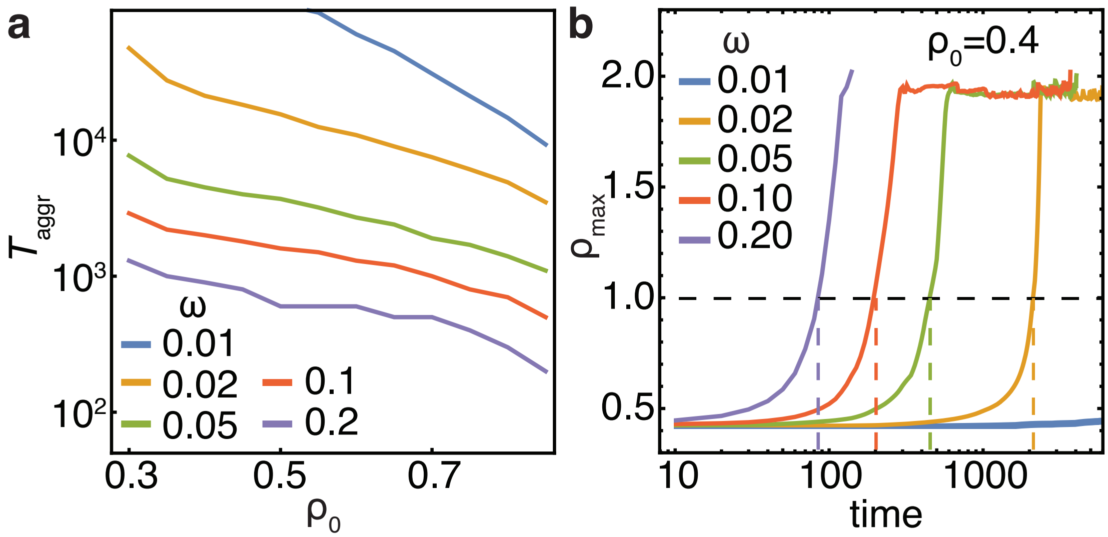

Supplementary Figure 1: Signaling-enhanced aggregation capabilities. a, Aggregation times $T_{\rm aggr}$ of the hydrodynamic system, main text Eqs. (6)-(9), reaching the polar-order transition at $\rho = 1$ from a homogeneous initial density ρ_0. We observe faster aggregation for higher initial densities as well as larger signaling susceptibilities ω. b, Corresponding temporal evolution of the system's maximum density $\rho_{\rm max}$ evolving from a homogeneous initial density $\rho_0 = 0.4$ for different values of ω. We determine the aggregation times $T_{\rm aggr}$ (dashed colored lines) as the first times at which the critical density (dashed black line) is reached, $\rho_{\rm max} = \rho_c = 1$. Other parameters as given in SI section 3.

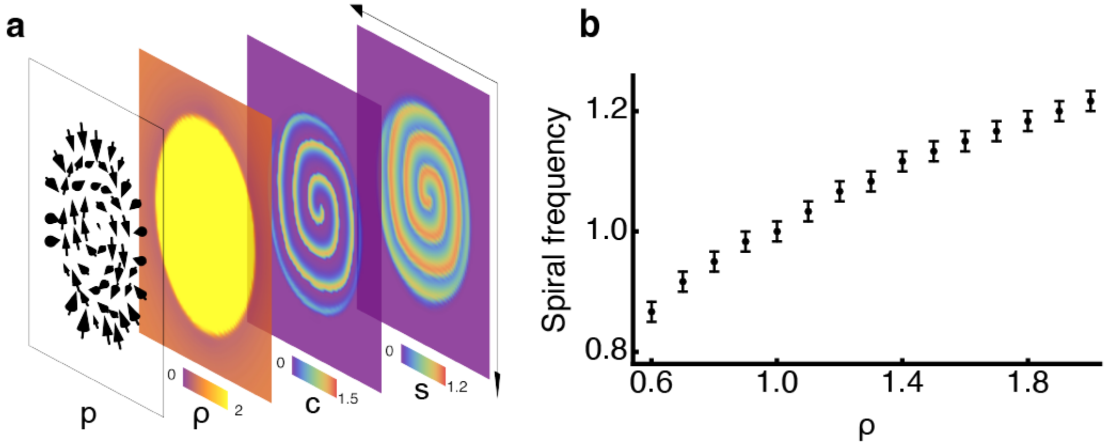

Supplementary Figure 2: Spiral waves and vortex solution in the hydrodynamic model. a, Vortex solution with persistent spiral wave activity in the hydrodynamic model, see Methods. The composite image containing layers representing the orientation vector field $\boldsymbol{p}(\boldsymbol{r})$ (arrows), the local density profile $\rho(\boldsymbol{r})$, concentration of signaling molecules $c(\boldsymbol{r})$, and field of state $s(\boldsymbol{r})$. b, Dependence of spiral frequency on spatially homogeneous density values ρ. Error bars indicate error ranges arising from the numerical measurement of spiral frequencies. Parameters as stated in SI section 3.

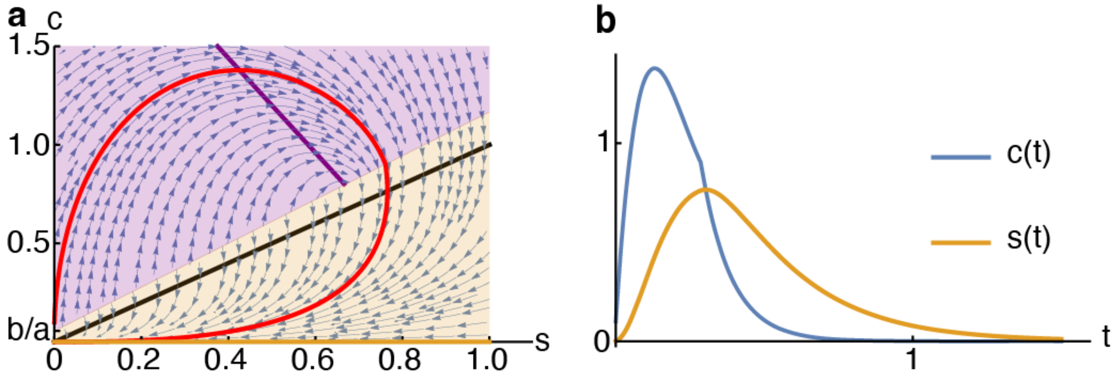

Supplementary Figure 3: Excitable dynamics of the well-mixed signaling system. The agents serve both as a source of chemical signals and can adapt their internal state to the chemical environment. In this process, the release of the chemicals by the agents depends on the internal state of the agents and the state of the environment. The combination of these factors leads to a 'sense-and-response' system that exhibits excitable dynamics. a, Phase-space flow of the excitable system, main text Eqs. (3), (5). The black line indicates the nullcline $c = s$ of the agents' state kinetics, main text Eq. (5). Due to the discontinuous switch in the agents' signal relaying capability, there are two nullclines (violet and orange) originating from the signaling kinetics, main text Eq. (3), with $c = \beta/\alpha(1-s)$ and $c = 0$, respectively. These nullclines are valid in the correspondingly colored areas $c \gtrless (s+b)/a$. The red trajectory highlights an excursion in phase space upon initial excitation. b, Dynamics of the chemical concentration c and the signaling state s corresponding to the red trajectory in a. Parameters as stated in SI section 3. Time is measured in the units of the decay rate $[\alpha]$.

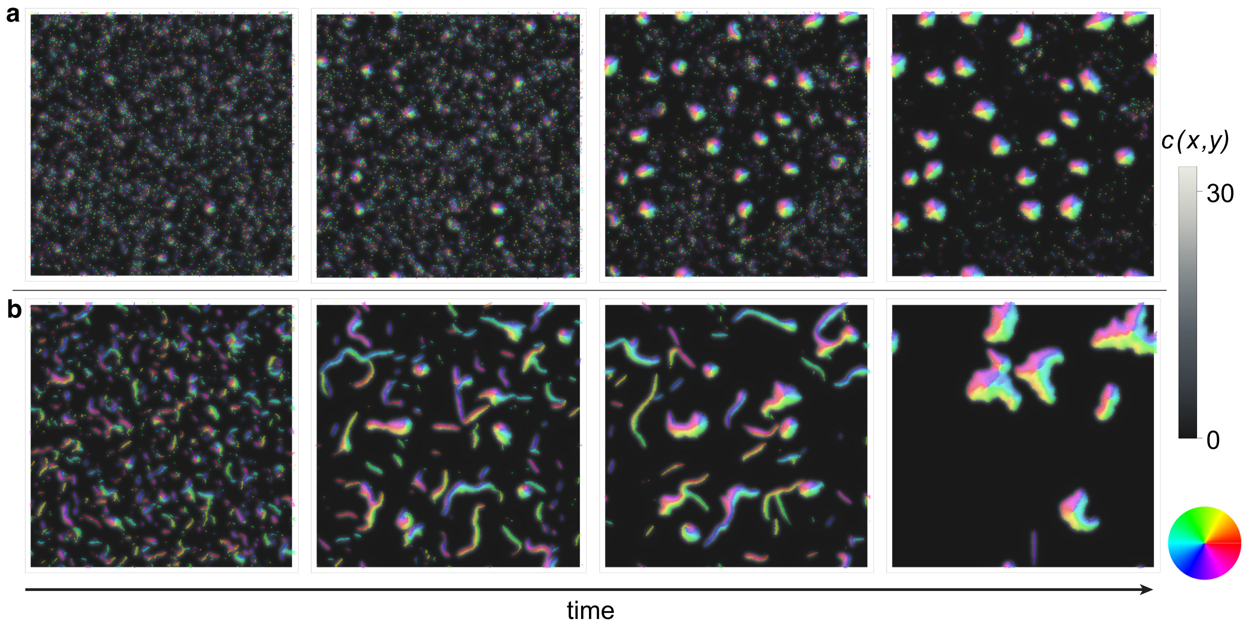

Supplementary Figure 4: Time evolution of a reduced model, lacking the internal decision making machinery of the self-propelling agents, main text Eqs. (1), (2), and SI Eq. (27). The two parameter regimes shown in panels a and b illustrate localized cluster formation as a generic form of aggregation in the model. The clusters exhibit an interface-controlled coarsening behavior. a, Formation of localized clusters for small polar alignment $\Gamma = 0.01$. b, Cluster formation with intermediate transient solutions for stronger polar alignment, $\Gamma = 0.1$. Agent colors indicate the polar orientation and background colors represents concentrations of the communication field $c(\boldsymbol{r}, t)$, see Eq. (27). Parameters as in table 2 with $r_p = 0.5$, $\beta = 0.9$, and $\omega = 0.05$.

Supplementary References

1. Aranson, I. S. & Tsimring, L. S. Pattern formation of microtubules and motors: Inelastic interaction of polar rods. Phys. Rev. E **71,** 050901 (2005).
2. Bertin, E., Droz, M. & Grégoire, G. Boltzmann and hydrodynamic description for self-propelled particles. Phys. Rev. E **74,** 022101 (2006).
3. Aranson, I. S. & Tsimring, L. S. Patterns and collective behavior in granular media: Theoretical concepts. Rev. Mod. Phys. **78,** 641 (2006).
4. Peshkov, A., Aranson, I. S., Bertin, E., Chaté, H. & Ginelli, F. Nonlinear field equations for aligning self-propelled rods. Phys. Rev. Lett. **109,** 268701 (2012).
5. Maryshev, I., Marenduzzo, D., Goryachev, A. B. & Morozov, A. Kinetic theory of pattern formation in mixtures of microtubules and molecular motors. Phys. Rev. E **97,** 22412 (2018).
6. Romanczuk, P., Erdmann, U., Engel, H. & Schimansky-Geier, L. Beyond the Keller-Segel model. Eur. Phys. J. Spec. Top. **157,** 61–77 (2008).
