# Supplementary material for: Multi-scale organization in communicating active matter
Source: Nat Commun. 2022 Nov 7;13:6727. doi: 10.1038/s41467-022-34484-2 (PMC9640622; doi:10.1038/s41467-022-34484-2)
Supplement: Supplementary file 2 — Description of Additional Supplementary Files [file 41467_2022_34484_MOESM2_ESM.pdf]

## Description of Additional Supplementary Files

**Supplementary Movie 1: Aggregation process in agent-based and hydrodynamic model.** Visual comparison of the aggregation process from a homogeneous disordered state in the agent-based model (left) and the hydrodynamic model (right). We observe a very similar behavior with an initial formation of smaller droplets that subsequently organize into streams. Particles collectively propagate in active droplets towards emerging vortices as organization centres with internal spiral wave activity. Parameters for the agent-based simulation are $N$=8,000, $\omega$=0.2. Parameters of the hydrodynamic model are $D_c$=2, $\varepsilon$=0.8, $\alpha$=2, $\beta$=8, $\sigma$=0.02 and remaining values as given in Supplementary Information section 3. Scale bars indicate a length of 10 units.

**Supplementary Movie 2: Droplets as predominant collective dynamic states.** Temporal dynamics of the density $\rho$ (left) and the signaling field $c$ (right) together with the local orientation **p** indicated by arrows. Results of numerical simulations of the hydrodynamic model accompanying Fig. 2.

**Supplementary Movie 3: Vortices as predominant collective dynamic states.** Temporal dynamics of the density $\rho$ (left) and the signaling field $c$ (right) together with the local orientation **p** indicated by arrows. Results of numerical simulations of the hydrodynamic model accompanying Fig. 2.

**Supplementary Movie 4: Rings as predominant collective dynamic states.** Temporal dynamics of the density $\rho$ (left) and the signaling field $c$ (right) together with the local orientation **p** indicated by arrows. Results of numerical simulations of the hydrodynamic model accompanying Fig. 2.

**Supplementary Movie 5: Silent bands as predominant collective dynamic states.** Temporal dynamics of the density $\rho$ (left) and the signaling field $c$ (right) together with the local orientation **p** indicated by arrows. Results of numerical simulations of the hydrodynamic model accompanying Fig. 2.

**Supplementary Movie 6: Streams as predominant collective dynamic states.** Temporal dynamics of the density $\rho$ (left) and the signaling field $c$ (right) together with the local orientation **p** indicated by arrows. Results of numerical simulations of the hydrodynamic model accompanying Fig. 2.

**Supplementary Movie 7: Polar bands with signaling activity as predominant collective dynamic states.** Temporal dynamics of the density $\rho$ (left) and the signaling field $c$ (right) together with the local orientation **p** indicated by arrows. Results of numerical simulations of the hydrodynamic model accompanying Fig. 2.

**Supplementary Movie 8: Hierarchical aggregation and information processing.** Time evolution of the density and signaling fields and corresponding information measures accompanying the data shown in Fig. 3.

**Supplementary Movie 9: Ring annihilation due to interaction with a vortex.** A stable vortex interacts with a meta-stable ring. Due course of time, the ring is destabilized by the interaction with the vortex and mass is transferred to the latter in a stream-like fashion. Parameters of the hydrodynamic simulation as detailed in Supplementary Methods section 3, with $\sigma$= 0.05.
